# Neuronal differentiation of human dental pulp stem cells induced by co-treatment of ergothioneine

**DOI:** 10.1371/journal.pone.0331120

**Published:** 2025-09-02

**Authors:** Amarin Thongsuk, Peeratchai Seemaung, Phetcharat Phanthong, Kajohnkiart Janebodin, Nisarat Ruangsawasdi, Thanasup Gonmanee, Chareerut Phruksaniyom, Tarinee Chodchavanchai, Tatcha Balit, Kenneth L. White, Charoensri Thonabulsombat, Anupong Thongklam Songsaad

**Affiliations:** 1 Department of Anatomy, Faculty of Science, Mahidol University, Bangkok, Thailand; 2 Department of Pharmacology, Faculty of Dentistry, Mahidol University, Bangkok, Thailand; 3 Department of Anatomy, Faculty of Dentistry, Mahidol University, Bangkok, Thailand; 4 Chakri Naruebodindra Medical Institute, Faculty of Medicine Ramathibodi Hospital, Mahidol University, Samut Prakarn, Thailand; 5 Department of Population Health and Pathobiology, College of Veterinary Medicine, North Carolina State University, Raleigh, North Carolina, United States of America; 6 School of Medicine, Walailak University, Nakhon Si Thammarat, Thailand; 7 Department of Animal Dairy and Veterinary Science, College of Agriculture and Applied Sciences, Utah State University, Logan, Utah, United States of America; Advanced Materials Technology Research Institute, National Research Centre, EGYPT

## Abstract

**Objective:**

Human dental pulp stem cells (hDPSCs) are promising adult stem cells that present multilineage differentiation ability. Interestingly, ergothioneine (ERGO) has the potential to uptake into the organic cation transporter N1 (OCTN1) to promote neuronal differentiation. Therefore, this study aims to demonstrate the effect of co-treatment of ergothioneine on the neuronal differentiation of hDPSCs.

**Methods:**

The hDPSCs were established from the impacted third molars. Subsequently, the hDPSCs investigated the cell viability with ergothioneine at concentrations of 0–500 µM for 30 hours. The non-cytotoxic concentration of ergothioneine was synergistically induced with the neuronal induction medium. The characteristics of differentiated cells were verified as neuronal cells (d-hDPSCs) by identification of the Nissl substance. The optimal concentration of ergothioneine, which triggered the highest neuronal differentiation of hDPSCs, was further confirmed by neuronal phenotypes via immunofluorescent staining, gene expression, and the ability of neurotransmitter release by intracellular calcium oscillation.

**Results:**

The isolated cells from human dental pulp tissue were characterized as mesenchymal stem cells (MSCs), verified as hDPSCs. The cellular toxicity of ergothioneine was not observed up to 500 µM for 30 hours. The d-hDPSCs presented a neuronal-like shape and positively expressed the Nissl substance. Interestingly, the highest number of neuronal-like cells was detected at 500 µM of ergothioneine. These neuronal-like cells exhibited the synaptic vesicle glycoprotein 2A (SV2A) expression and dynamic change of intracellular Ca^2+^, suggesting potential functional neuronal characteristics. Furthermore, co-treatment of ergothioneine at 500 µM triggered neurogenic maturation by decreasing Nestin and *NES* expression and increasing Beta-III tubulin, *TUBB3*, and microtubule-associated protein 2 (MAP2) expression, respectively.

**Conclusion:**

Co-treatment of ergothioneine at 500 µM can enhance neuronal differentiation, which has the potential to promote neurogenic maturation. Therefore, these findings suggest the alternative of using hDPSCs and the potential of ergothioneine co-treatment as stem cell-based therapy for further transplantation to cure various neurological diseases.

## Introduction

Neurological disorders of the central nervous system (CNS), which are defined as the Global Burden of Disease, cause permanent disability and global mortality [1]. The abnormalities are mainly caused by the loss of function of neuronal cells [2]. Moreover, the functional neuronal cells are fully differentiated cells that rarely present the self-renewal ability to regenerate themselves. Interestingly, adult neurogenesis is the dynamic process of generating functional neurons from their progenitor, neural stem cells (NSCs), when the CNS is damaged [3]. However, endogenous restoration by repairing with their progenitor cells is still limited [4]. Therefore, exogenous NSCs transplantation could be a potential approach to regenerate and replace the damaged neurons, leading to improving the quality of a patient’s life.

The establishment of NSCs can be isolated from the fetal brain and adult brain, which is localized at the subventricular zone of the lateral ventricle and the subgranular zone of the hippocampal formation of the dentate gyrus [5]. However, this method has serious ethical concerns regarding the invasive establishment and donor site morbidity, which causes damage to the donor at the isolated site and terminates the embryo’s life [6]. Moreover, isolating and culturing were difficult, in addition to the low number of NSCs [7]. Therefore, a potential alternative cell source that provides a high neurogenic potency with low donor site morbidity and low ethical considerations could be investigated to overcome these limitations.

Human dental pulp stem cells (hDPSCs) were discovered in the dental pulp tissue of the permanent teeth [8]. These cells have been characterized as mesenchymal stem cells (MSCs) according to plastic adherent ability, typical fibroblast-like morphology, expression of MSCs surface markers, and multilineage differentiation [9]. Moreover, the hDPSCs were identified as ectomesenchyme due to their origin from migratory neural crest stem cells, which exhibited and committed superior neurogenic potency [10]. Furthermore, dental pulp tissue is considered dental waste, which is obtained from tooth extraction. Also, isolation of hDPSCs can be performed with an easy-accessible method, low donor site morbidity, and few ethical concerns [11]. However, the efficiency of neuronal differentiation of hDPSCs is still has a low efficiency of differentiated cells [12,13]. Therefore, exogenous enhancers should be investigated to increase neuronal differentiation ability.

Recently, pharmaceutical herbal-extracted compounds have become of interest as an alternative potential source of new therapeutic agents for neurodegenerative disease [14]. Ergothioneine (ERGO) is a sulfur-containing amino acid, that is commonly found in mushroom species [15]. Interestingly, ergothioneine appears to be taken into cells through the organic cation transporter N1 (OCTN1) to regulate neuronal differentiation [16], neurogenesis [17], and microglial activation [18]. Furthermore, *in vivo* administration of ergothioneine can pass through the blood-brain barrier (BBB) [19], which indicates its regenerative potential in the CNS.

Recently, the expression of the OCTN1 receptor was discovered in mouse neural stem cells, which demonstrated the enhancing potential of ergothioneine for specific differentiation into the neuronal cell lineage [20]. Moreover, the hDPSCs were defined as the potential ectomesenchymal stem cells, which exhibited neuronal differentiation ability [12]. However, there are no investigations on enhancing the neuronal differentiation of hDPSCs with ergothioneine treatment. To address this gap, this study aims to demonstrate the potential of co-treatment with ergothioneine in enhancing the neuronal differentiation of hDPSCs for further transplantation in neurological disorders and hypothesize that ergothioneine could be used as an enhancer to promote neuronal differentiation of hDPSCs.

## Materials and methods

### Materials

Ergothioneine (L-(+)-Ergothioneine, purity ≥ 98%, Sigma-Aldrich, MI, USA).

### Isolation and culture of hDPSCs

The human-impacted third molars were collected from Thai patients (18–21 years old, n = 3) at the Oral and Maxillofacial Surgery Clinic, Dental Hospital, Faculty of Dentistry, Mahidol University, Bangkok, Thailand. The research protocol and ethical considerations were approved by the Ethics Review Committee for Human Rights Related to Human Experimentation of the Faculty of Dentistry/Faculty of Pharmacy, Mahidol University, Thailand (COE.No.MU-DT/PY-IRB 2021/019.2106). The procedure was conducted in accordance with the Declaration of Helsinki. The criteria of the teeth include the presence of dental pulp tissue, caries-free, no sign of trauma, pulp necrosis, or periodontal disease. Written informed consent was obtained for the experiment with all human subjects during the recruitment period (21 June 2021–21 June 2023). An enzymatic-disaggregation method was performed to isolate the cells from the dental pulp tissue [12]. Briefly, the teeth were maintained in a proliferating medium, which consisted of Minimum Essential Medium (MEM, Gibco, Life Technologies, NY, USA) supplemented with 10% fetal bovine serum (FBS, Gibco, Life Technologies), and 1% antibiotic-antimycotic (Gibco, Life Technologies). The teeth were extracted for dissecting dental pulp tissue. Then, the dissected pulp tissue was digested using enzymatic digestion with collagenase type I (Worthington, NJ, USA) and Dispase II (Sigma-Aldrich) for 1 hour at 37°C. Consequently, the sample was filtered with a 70 µm cell strainer (Falcon, Fisher Scientific, MA, USA). Then, the cells were seeded into a culture vessel (T-75 cm^2^ flask, Nunc, Thermo Scientific, MA, USA). The cells were cultured in the proliferating medium at 37°C, 5% CO_2_, and 95% humidity incubator. After the cell population grows to 80% confluence, a subculture was performed by 0.05% trypsin ethylenediaminetetraacetic acid (EDTA) (Gibco, Life Technologies) to expand the population. Cells in passages 3–6 were used for this study.

### Characterization of MSCs properties

The isolated cells were seeded at the density of 2x10^4^ cells/well into 24-well plates (Nunc_,_ Thermo Scientific) and cultured with the proliferating medium to 80% confluence. The property of ectomesenchymal origin was investigated by immunofluorescence staining of Nestin and Beta-III tubulin.

To investigate osteogenic differentiation ability, the isolated cells were seeded at the density of 2x10^4^ cells/well into 24-well plates. After culturing with the proliferating medium, until they reached 80% confluence, the cells were induced by an osteogenic induction medium for 3 weeks. The osteogenic induction medium consists of MEM supplemented with 10% FBS, 1% antibiotic-antimycotic, 10 mM Beta-glycerophosphate (Sigma-Aldrich), 0.1 µM dexamethasone (Sigma-Aldrich), and 50 mg/ml ascorbate-2-phosphate (Sigma-Aldrich). The induction medium was changed every 2 days. After completing the induction, the osteogenic differentiation was observed in the calcification of the extracellular matrix using Alizarin red staining.

To investigate adipogenic differentiation ability, the isolated cells were seeded at the density of 2x10^4^ cells/well into 24-well plates. After they reached 80% confluence, the cells were induced by an adipogenic induction medium for 3 weeks. The adipogenic induction medium consists of MEM supplemented with 10% FBS, 1% antibiotic-antimycotic, 0.5 mM 3-isobutyl-1-methylxanthine (Sigma-Aldrich), 50 µM indomethacin (Sigma-Aldrich), 1 µM dexamethasone, and 1 µg/mL insulin (Sigma-Aldrich). The induction medium was changed every 2 days. After completing the induction, the adipogenic differentiation was detected in the lipid droplets using Oil Red O staining.

To investigate neurogenic differentiation ability, the isolated cells were seeded at the density of 2x10^4^ cells/well into 24-well plates and cultured with the proliferating medium until they reached 80% confluence. Then, neuronal differentiation was induced by the two phases of the neuronal induction medium. First, the cells were cultured in the neuronal induction medium phase I, which consisted of Dulbecco’s Modified Eagle Medium: Nutrient Mixture F-12 (DMEM/F-12, Gibco, Life Technologies) supplemented with 10% FBS, 1% antibiotic-antimycotic, 500 µM Beta-mercaptoethanol (Sigma-Aldrich), and 10 ng/mL basic fibroblast growth factor (bFGF, Gibco, Life Technologies) for

**Fig 1:**
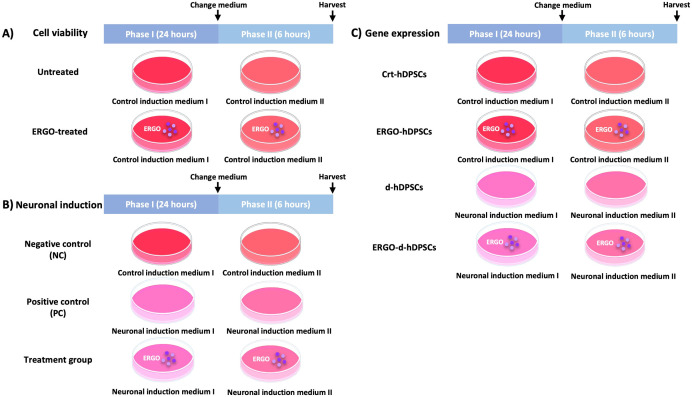
Experimental groups, components of the culture media, and treatment period. The scheme represents the experimental groups, components of culture media, and treatment period. The experiments were performed within 2 phases, including phase I for 24 hours and phase II for 6 hours. **(A)** Cell viability procedures consisted of 2 groups, including the untreated and ERGO-treated groups. **(B)** The neuronal induction procedure is divided into 3 groups, including negative control (NC), positive control (PC), and the treatment group. **(C)** Gene expression is composed of 4 groups such as Crt-hDPSCs, ERGO-hDPSCs, d-hDPSCs, and ERGO-d-hDPSCs.

24 hours. Consequently, the cells were incubated with phase II neuronal induction medium that consisted of DMEM/F-12 supplemented with 2% dimethyl sulfoxide (DMSO, Sigma-Aldrich), 1% antibiotic-antimycotic, and 100 µM butylated hydroxyanisole (BHA, Sigma-Aldrich) for 6 hours (Table 1 and Fig 1B). After completing the induction, the neurogenic differentiation was detected in neuronal cell-like morphology and positively stained by Cresyl violet staining.

**Table 1:** Experimental groups, components of the culture media, and treatment period.

Experimental groups	Phase I (24 hours)	Phase II (6 hours)
**1. Cell viability**		
**Untreated**	**Control induction medium phase I** • DMEM/F-12 • 10% FBS • 1% Antibiotic-Antimycotics	**Control induction medium phase II** • DMEM/F-12 • 1% Antibiotic-Antimycotics
**ERGO treated**	**Control induction medium phase I** • DMEM/F-12 • 10% FBS • 1% Antibiotic-Antimycotics **with ergothioneine supplement** • 10, 25, 50, 100, 250, 500 μM ERGO	**Control induction medium phase II** • DMEM/F-12 • 1% Antibiotic-Antimycotics **with ergothioneine supplement** • 10, 25, 50, 100, 250, 500 μM ERGO
**2. Neuronal induction**		
**Negative control** **(NC)**	**Control induction medium phase I** • DMEM/F-12 • 10% FBS • 1% Antibiotic-Antimycotics	**Control induction medium phase II** • DMEM/F-12 • 1% Antibiotic-Antimycotics
**Positive control** **(PC)**	**Neuronal induction medium phase I** • DMEM/F-12 • 10% FBS • 1% Antibiotic-Antimycotics • 10 ng/ml bFGF • 500 μM beta-mercaptoethanol	**Neuronal induction medium phase II** • DMEM/F-12 • 1% Antibiotic-Antimycotics • 2% DMSO • 100 μM BHA
**Treatment group**	**Neuronal induction medium phase I** • DMEM/F-12 • 10% FBS • 1% Antibiotic-Antimycotics • 10 ng/ml bFGF • 500 μM beta-mercaptoethanol **with ergothioneine supplement** • 10, 25, 50, 100, 250, 500 μM ERGO	**Neuronal induction medium phase I** • DMEM/F-12 • 1% Antibiotic-Antimycotics • 2% DMSO • 100 μM BHA **with ergothioneine supplement** • 10, 25, 50, 100, 250, 500 μM ERGO
**3. Gene expression**		
**Crt-hDPSCs**	**Control induction medium phase I** • DMEM/F-12 • 10% FBS • 1% Antibiotic-Antimycotics	**Control induction medium phase II** • DMEM/F-12 • 1% Antibiotic-Antimycotics
**ERGO-hDPSCs**	**Control induction medium phase I** • DMEM/F-12 • 10% FBS • 1% Antibiotic-Antimycotics **with ergothioneine supplement** - 500 μM ERGO	**Control induction medium phase II** • DMEM/F-12 • 1% Antibiotic-Antimycotics **with ergothioneine supplement** - 500 μM ERGO
**d-hDPSCs**	**Neuronal induction medium phase I** • DMEM/F-12 • 10% FBS • 1% Antibiotic-Antimycotics • 10 ng/ml bFGF • 500 μM beta-mercaptoethanol	**Neuronal induction medium phase II** • DMEM/F-12 • 1% Antibiotic-Antimycotics • 2% DMSO •100 μM BHA
**ERGO-** **d-hDPSCs**	**Neuronal induction medium phase I** • DMEM/F-12 • 10% FBS • 1% Antibiotic-Antimycotics • 10 ng/ml bFGF • 500 μM beta-mercaptoethanol **with ergothioneine supplement** • 500 μM ERGO	**Neuronal induction medium phase I** • DMEM/F-12 • 1% Antibiotic-Antimycotics • 2% DMSO • 100 μM BHA **with ergothioneine supplement** • 500 μM ERGO

Consequently, the analysis of cell surface markers was performed using a BD FACS Canto Flow cytometer (BD Biosciences, CA, USA) and analyzed by BD FACSDiva software (BD Biosciences). The isolated cells (1x10^6^ cells) were collected and analyzed by specific MSC markers, including anti-human CD73 (Biolegend, CA, USA), anti-human CD90 (Biolegend), anti-human CD105 (Biolegend), and anti-human CD146 (Biolegend). The hematopoietic stem cells (HSCs) marker, using anti-human CD34 (Biolegend) was used as the negative control.

Finally, the isolated cells were seeded at a density of 500 cells/well into a 6-well plate (Nunc_,_ Thermo Scientific) and cultured in the proliferating medium for 12 days. Every 2 days, the proliferating medium was changed. Then, the colonies were visualized by Giemsa staining.

### Cell viability of ergothioneine-treated hDPSCs

The colorimetric assay with methylthiazolyldiphenyl-tetrazolium bromide (MTT, Sigma-Aldrich) was performed to determine the cellular viability of the ergothioneine-treated hDPSCs. The stock of ergothioneine solution was freshly prepared and diluted with the culture medium. The characterized hDPSCs were seeded at the density of 5x10^4^ cells/well into a 96-well plate (Nunc_,_ Thermo Scientific) and cultured with the proliferating medium for 24 hours. Then, the hDPSCs were cultured with the control induction medium phase I, which consisted of DMEM/F-12, 10% FBS, 1% antibiotic-antimycotic, and ergothioneine at various concentrations (0, 10, 25, 50, 100, 250, and 500 µM) for 24 hours. Consequently, the cells were incubated with control induction medium phase II, which consisted of DMEM/F-12, 1% antibiotic-antimycotic, and ergothioneine at various concentrations (0, 10, 25, 50, 100, 250, and 500 µM) for 6 hours (Table 1 and Fig 1A). Then, 0.5 mg/mL MTT solution was added and incubated at 37°C for 2 hours. Consequently, the solution was removed and solubilized with DMSO to dissolve the crystal formazan. Finally, the absorbance was measured at 570 nm (soluble formazan) and 690 nm (background) by using a microplate reader (Epoch, Fisher Scientific, MA, USA). The percentage of cell viability of ergothioneine-treated hDPSCs was reported following this formula:


$$\text{Cellviability}(\%)=\frac{\text{MeanODoftreatedgroup}(\text{OD570}-\text{OD690})}{\text{MeanODofcontrolgroup}(\text{OD570}-\text{OD690})}\times 100$$

### Co-treatment of ergothioneine with neuronal induction

The hDPSCs were seeded at the density of 1x10^5^ cells/well into 6-well plates and cultured with the proliferating medium for 24 hours. The negative control (NC) was performed by incubation with the control induction medium phase I for 24 hours. Then, the medium was replaced with the control induction medium phase II for 6 hours. The positive control (PC) was performed by incubation with the neuronal induction medium phase I for 24 hours. Then, the medium was replaced with the neuronal induction medium phase II for 6 hours. In the treatment group, the cells were incubated with 2 phases of neuronal induction medium with non-cytotoxic concentrations of ergothioneine. The neuronal induction medium phase I was supplemented with ergothioneine at various non-cytotoxic concentrations for 24 hours. Consequently, the medium was changed into the neuronal induction medium phase II, which was supplemented with ergothioneine at various non-cytotoxic concentrations for 6 hours (Table 1 and Fig 1B).

### Characterization of neuronal-like cells

After neuronal induction, the differentiated cells were observed by the bright-field microscope. Cell imaging was randomly captured in 5 areas/well by the compact cell culture microscope (Olympus, Hamburg, Germany). The differentiated cells, which presented the neuronal-like appearance, and were quantified into the percentage of neuronal-like cells.

Moreover, Cresyl violet staining was performed to further verify the neuronal-like cells by identification of the Nissl substance, which is a characteristic of neurons. Cell imaging was randomly captured in 5 areas/well by the compact cell culture microscope. The differentiated cells, which presented neuronal-like morphologies and intense Nissl substance, were defined as the Cresyl violet positive cells and quantified into the percentage of Cresyl violet positive cells.

To determine the potential of ergothioneine synergistic treatment in enhancing neuronal differentiation of the hDPSCs, the concentration of ergothioneine synergistic treatment, which triggers the highest neuronal differentiation ability of the hDPSCs, represents the optimal condition. These neuronal-like cells were termed “ERGO-d-hDPSCs” and were further investigated the neuronal profiling.

### Investigation of the ergothioneine’s optimal condition on neuronal differentiation

The ERGO-d-hDPSCs were further investigated in several neuronal profiles. The expression of Beta-III tubulin was demonstrated to identify the neuronal appearances and quantify the percentage of neuronal-like cells. Moreover, the neuronal stage of the differentiated cells was performed by Nestin immunofluorescence staining to indicate the early neuronal stage and Beta-III tubulin and MAP2 immunofluorescence staining to indicate the late neuronal stage. The fluorescent intensity of Nestin, Beta-III tubulin, and MAP2 was investigated to demonstrate the potential of ergothioneine co-treatment of the neuronal differentiation ability of hDPSCs. Furthermore, the expression level of neuronal-associated genes and ergothioneine transporter (*SLC22A4*) was demonstrated by Quantitative real-time reverse transcription polymerase chain reaction (qRT-PCR).

### Functional neuronal activity

The expression of SV2A was investigated to indicate the potential of functional neuronal activity. The Crt-hDPSCs (NC), d-hDPSCs (PC), and ERGO-d-hDPSCs were observed the qualified expression of SV2A. Furthermore, the neurotransmitter-releasing activity of neuronal cells was demonstrated by intracellular calcium oscillation. The Crt-hDPSCs were used as the control.

### Identification of Nissl substance by Cresyl violet staining

The samples were fixed in 4% paraformaldehyde (Sigma-Aldrich) for 1 hour, then the cells were washed with phosphate-buffered saline (PBS, Sigma-Aldrich) for 5 minutes 2 times and double-distilled water for 1 minute. After that, the cells were stained with Cresyl violet solution (Electron Microscopy Sciences, PA, USA) for 1 hour under dark conditions. Then, the cells were serially dehydrated with 90%, 95%, and 100% ethanol (MERCK, Darmstadt, Germany), respectively. The Nissl substance was revealed by Cresyl violet staining. Verifying neuronal cells in the differentiated cells was defined by exhibiting a neuronal-like shape and positively stained Nissl substance.

### Immunocytochemistry

The samples were fixed in 4% paraformaldehyde for 1 hour. Then incubated with 20% ice-cold methanol (MERCK) in PBS for 5 minutes and permeabilized with 0.5% Triton X-100 (Sigma-Aldrich) in PBS overnight at 4°C. Then, the samples were blocked with 15% bovine serum albumin (BSA, Sigma-Aldrich) at 4°C for 12 hours. The samples were incubated with 1:500 anti-mouse Nestin antibody (Biolegend), 1:1,000 anti-mouse Beta-III tubulin antibody (Biolegend), 1:200 anti-rabbit MAP2 antibody (Biolegend), and 1:50 anti-mouse SV2A antibody (Santa Cruz Biotechnology, TX, USA), which were diluted with 5% BSA in PBS with 0.05% Tween-20 (Sigma-Aldrich) overnight at 4°C. Then, the samples were conjugated with 1:1,000 goat anti-mouse conjugated Alexa Fluor plus 488 secondary antibodies (Invitrogen, NY, USA) and 1:1,000 donkey anti-rabbit conjugated Alexa Fluor plus 594 secondary antibodies (Invitrogen) for 4 hours at room temperature. Nuclei were counter-stained and mounted with Prolong Diamond Antifade Mountant with DAPI (Invitrogen). The immunofluorescence staining was captured under the confocal microscope platforms STELLARIS 5 (Leica Microsystems, Wetzlar, Germany). Cell imaging and measurement of fluorescent intensity were interpreted with the software Leica Application Suite X version 4.2.1.23810 (Leica Microsystems).

### Quantitative real-time reverse transcription polymerase chain reaction (qRT-PCR)

To investigate the neurogenic-associated marker in mRNA expression level, the experimental groups, including crt-hDPSCs, ERGO-hDPSCs, d-hDPSCs, and ERGO-d-hDPSCs, were performed (Table 1 and Fig 1C). Total RNA was extracted using a RiboEx^TM^ kit (GeneAll Biotechnology, Seoul, South Korea) and converted into cDNA using the revert aid first-strand cDNA synthesis kit (Thermo Scientific). The qRT-PCR was performed using KAPA SYBR FAST qPCR kits (Sigma-Aldrich) with a CFX97 touch real-time PCR detection system (Bio-Rad). The qRT-PCR conditions were 95°C for 3 minutes, followed by 40 cycles of 95°C for 3 seconds and 63.3°C for 30 seconds. The interested primers (Integrated DNA Technologies, Gemini Singapore Science Park II, Singapore) used in this study are listed in Table 2. The glyceraldehyde 3-phosphate dehydrogenases (*GAPDH*) was used as an internal control, the expression of the interested genes was measured by 2^-**ΔΔ**CT^ [21].

**Table 2:** Forward and reverse primers for qRT-PCR.

Genes	Primers	Sequences (5’-3’)	References
*NES*	Forward	AGG AAA AGA CCA TCT GCC CG	NM_006617.2
	Reverse	TCC TTT GCC ACA CCC CTT TT	
*TUBB3*	Forward	CAA CCA GAT CGG GGC CAA GTT	NM_006086.4
	Reverse	GAG GCA CGT ACT TGT GAG AAG A	
*MAP2*	Forward	CAG AGG AGG TGT CTG CAA GG	NM_002374.4
	Reverse	TCA GCT GCT AAA GGC AGA GC	
*SV2A*	Forward	CTG GCT TAG CCT CCC AAT CT	NM_014849.5
	Reverse	GCC AAT GAG TGC CTA GGA AGG	
*SLC22A4*	Forward	CAA CGC CTT CAG CCT GTT TC	NM_003059.3
	Reverse	CTA CGG GTG ATG ACA GCG TT	
*GAPDH*	Forward	CTGACTTCAACAGCGACACC	NM_002046.7
	Reverse	TGCTGTAGCCAAATTCGTTG	

### Intracellular calcium oscillation

To demonstrate the functional neuronal activity, the intracellular calcium oscillation was performed to indicate neurotransmitter release in differentiated cells. The specimens were incubated with DMEM/F12, 1% antibiotic-antimycotic, 0.08% Pluronic acid (Invitrogen), and 3 µM Fluo-3 AM (Invitrogen) at 37°C for 1 hour. Then, specimens were washed with DMEM/F12, 1% antibiotic-antimycotic, and PBS, respectively. Consequently, the specimens were maintained with Tyrode’s solution, which consisted of 1 mM MgCl_2_ (Sigma-Aldrich), 2 mM CaCl_2_ (Sigma-Aldrich), 5 mM KCl (Sigma-Aldrich), 25 mM HEPES (Gibco, Life Technologies), 30 mM glucose (Sigma-Aldrich), and 129 mM NaCl (Sigma-Aldrich). The 50 mM KCl was used to stimulate the neurotransmitter-releasing ability of the differentiated cells. The Crt-hDPSCs were used as the negative control. The fluorescent intensity of calcium ions was recorded in time-lapse at 506 nm for120 seconds by the Confocal Microscope Platforms STELLARIS 5 and interpreted using the software Leica Application Suite X version 4.2.1.23810.

### Statistical analysis

The experiments were repeated 3 times. The data were expressed as mean ± standard error of the mean (SEM). One-way ANOVA and Tukey’s multiple comparison tests were used to compare the differences between the Crt-hDPSCs, the d-hDPSCs, and the ERGO-d-hDPSCs. The independent sample *t*-tests were used to compare the difference in gene expression by GraphPad Prism. The differences with *p-value < 0.05, **p-value < 0.01, or ***p-value < 0.001 were considered significant.

## Results

### Characterization of hDPSCs

**Fig 2:**
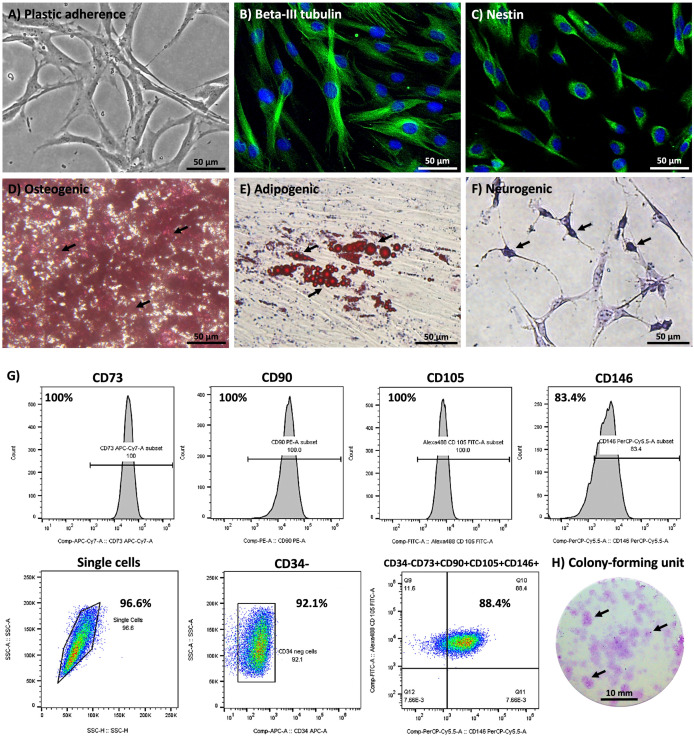
Characterization of hDPSCs. **(A)** The isolated cells can grow on plastic adherent culture vessels, revealing the typical fibroblast-like shape morphology. **(B-C)** The ectomesenchyme origin was demonstrated with Beta-III tubulin (green) and Nestin (green) staining, respectively. The nuclei were counter-stained with DAPI (blue). **(D-F)** Multipotential differentiation abilities were demonstrated through osteogenic (black arrows indicate the calcified nodules), adipogenic (black arrows indicate lipid droplets), and neurogenic (black arrows indicate Nissl bodies). **(G)** The isolated cells positively expressed MSCs markers. These cells that negatively expressed CD34 and co-expressed CD34-, CD73 + , CD90 + , CD105 + , and CD146 + represented the major population. **(H)** The isolated cells form colonies, which were positively stained with Giemsa dye (black arrows indicate colonies). Scale bars: A-F = 50 µm, and H = 10 mm.

The isolated cells had a fibroblast-like morphology and were able to grow on culture vessels to represent plastic adherence ability (Fig 2A). Without administration of neurogenic induction, immunofluorescence staining confirmed that the cells presented the ectomesenchymal origin, as they positively stained for Beta-III tubulin (Fig 2B) and Nestin (Fig 2C). Additionally, under optimal differentiation-inducing conditions, the cells were able to differentiate into osteocytes, adipocytes, and neuronal cells. These differentiated cells demonstrated the extracellular calcified nodules (Fig 2D), accumulation of lipid droplets (Fig 2E), and exhibited Nissl substance (Fig 2F) as revealed through Alizarin red, Oil Red O, and Cresyl violet staining, respectively. These results indicated that the isolated cells demonstrated the multilineage differentiation ability. Moreover, flow cytometry profiling of the cell surface antigen molecules of these cells demonstrated high expression of positive markers for MSCs, including CD73, CD90, CD105, and CD146, as indicated by the high intensity and cell count of histograms. Moreover, these cells that negatively expressed CD34 and co-expressed CD34-, CD73 + , CD90 + , CD105 + , and CD146 + were a major population (Fig 2G). Finally, the isolated cells can form colonies with positively stained Giemsa dye, indicating their self-renewal ability (Fig 2H). Taken together, the isolated cells-derived human dental pulp tissue exhibiting the properties of MSCs, were verified as hDPSCs.

### Cell viability of ergothioneine-treated hDPSCs

**Fig 3:**
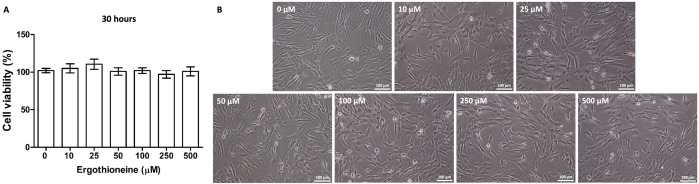
Cellular viability of ergothioneine-treated hDPSCs. **(A)** The cells were incubated with 0, 10, 25, 50, 100, 250, and 500 µM ergothioneine for 30 hours. Cellular toxicity was not observed at any concentration. **(B)** The ergothioneine-treated hDPSCs (0-500 µM) presented the typical fibroblast-like morphology. The data were presented as mean ± SEM, n = 3. Scale bars: B = 100 µm.

To investigate the cytotoxicity of ergothioneine on hDPSCs viability, the hDPSCs were incubated with 0, 10, 25, 50, 100, 250, and 500 µM of ergothioneine for 2 phases of treatment (30 hours), and performed the colorimetric assay by MTT. The viability of the cells was not adversely impacted by any of the concentrations of ergothioneine used for 30 hours period (Fig 3A). IC_50_ was reported as more than 500 µM. Furthermore, the cell morphology of ergothioneine-treated hDPSCs (10–500 µM) presented the typical fibroblast-like shape (Fig 3B) that was similar to the untreated cells (0 µM) and the primary hDPSCs (Fig 2A).

### Synergistic effect of ergothioneine on neuronal induction

**Fig 4:**
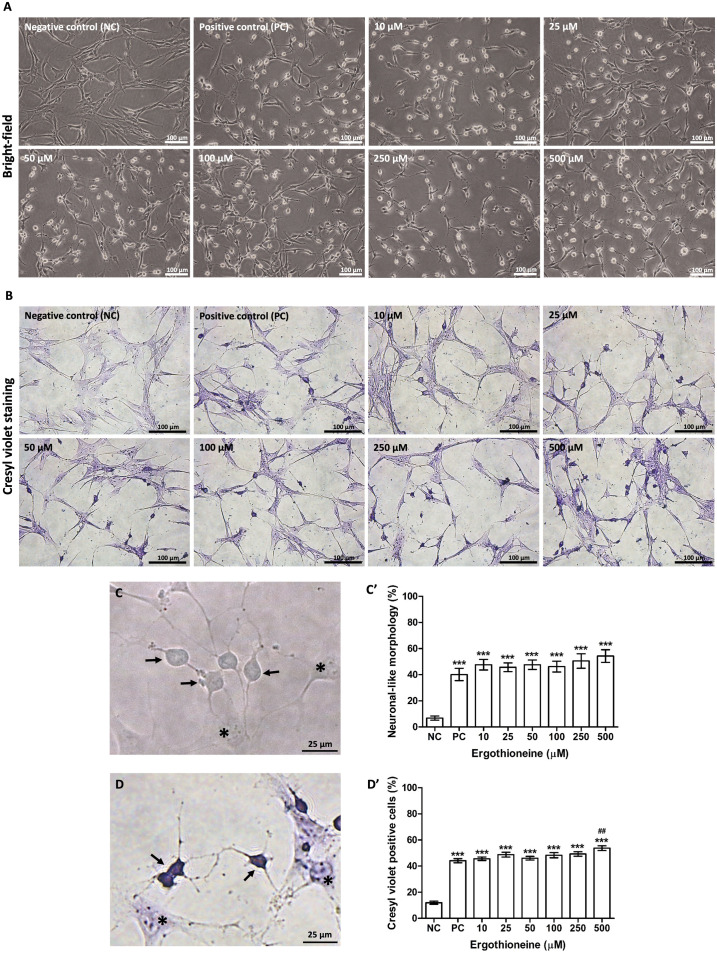
The co-treatment of ergothioneine on neuronal induction. **(A)** The cells were incubated in the neuronal induction medium and synergistically treated with non-cytotoxic concentrations of ergothioneine for 30 hours. The bright-field microscopic images demonstrated that neuronal-like cells were observed at the positive control (PC) and various concentrations of ergothioneine, whereas the majority cell population in the negative control (NC) presented the typical fibroblast-like morphology. **(B)** The Cresyl violet staining revealed that the neuronal-like cells positively detected the Nissl substance. **(C)** The high magnification of differentiated cells (black arrows) and undifferentiated cells (black asterisks) was revealed by bright field imaging. **(C’)** The percentage of neuronal-like cells showed an increasing pattern in the positive control and the ergothioneine treatment group. **(D)** The high magnification of Cresyl violet-positive cells was detected in the neuronal-like cells as dark purple-stained (black arrows). The undifferentiated cells only presented the pale purple-stained nuclei and dark spots of nucleoli (black asterisks). **(D’)** The highest percentage of Cresyl violet-positive cells was observed in the 500 µM ergothioneine. The data were expressed as mean ± SEM, ***p-value < 0.001 when compared to the negative control, ^##^*p*-value < 0.01 when compared to the positive control, Tukey’s Multiple Comparison Test, n = 3. Scale bars: A, B = 100 µm, and C, D = 25 µm.

The non-cytotoxic concentrations of ergothioneine were synergistically incubated with the neuronal induction medium. The bright field microscopy demonstrated that the differentiated cells exhibited round cell bodies and neuronal processes as the neuronal-like morphology (Fig 4C, black arrows) and were observed in the positive control (PC) and the ergothioneine treatment group. In contrast, the negative control (NC) presented the undifferentiated cells, revealing the fibroblast-like morphology (Fig 4C, black asterisks), as the majority population (Fig 4A). The percentage of the neuronal-like cells significantly showed an increasing pattern in the ergothioneine treatment group as the concentrations were increased (Fig 4C’). The Cresyl violet staining indicated the Nissl substance, which was the typical characteristic of the neuronal-like cells in the positive control and the ergothioneine treatment group (Fig 4B). Moreover, the high magnification of Cresyl violet imaging indicated that the neuronal-like cells positively expressed the Nissl body as the dark purple staining (Fig 4D, black arrows). Whereas identification of this Nissl body was not presented in the undifferentiated cells (Fig 4D, black asterisks). The percentage of Cresyl violet-positive cells (neuronal-like cells) in the positive group was significantly increased when compared with the negative control. The ergothioneine treatment enhanced the neuronal differentiation of the hDPSCs, resulting in a significant increase in the percentage of Cresyl violet-positive cells when compared with the negative control and positive control, respectively. Importantly, the highest neuronal differentiation ability was observed at ergothioneine 500 µM treatment and served as the optimal condition (Fig 4D’). Their neuronal cells were termed “ERGO-d-hDPSCs” and further investigated the neuronal profiling.

### Number of neuronal-like cells and neuronal types of ergothioneine co-treatment

**Fig 5:**
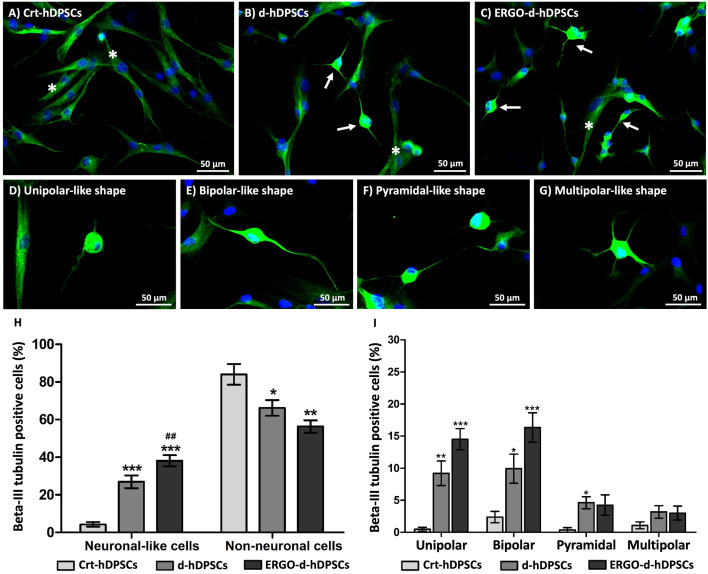
The number of neuronal-like cells and neuronal types of ergothioneine co-treatment. **(A-C)** The neuronal-like cells were positively expressed Beta-III tubulin (green) with neuronal-like morphology at the d-hDPSCs and the ERGO-d-hDPSCs (white arrows), while the undifferentiated cells presented the fibroblast-like morphology (white asterisks). The nuclei were counter-stained with DAPI (blue). **(D-G)** The several neuronal-like appearances were revealed as unipolar, bipolar, pyramidal, and multipolar, respectively. **(H)** The highest Beta-III tubulin-positive cells with neuronal-like morphology were observed at the ERGO-d-hDPSCs. **(I)** The potential of ergothioneine co-treatment promotes neuronal differentiation of hDPSCs into neuronal-like cells, which are specific to the unipolar-like and bipolar-like shapes. The data were expressed as mean ± SEM, *p-value < 0.05, **p-value < 0.01, ***p-value < 0.001 when compared to the Crt-hDPSCs, ^##^*p*-value < 0.01 when compared to the d-hDPSCs, Tukey’s Multiple Comparison Test, n = 3. Scale bars: A-G = 50 µm.

To demonstrate the neuronal profiling of ergothioneine co-treatment, the ERGO-d-hDPSCs were investigated the neuronal appearances by immunophenotyping to quantify the percentage of neuronal-like cells. The ERGO-d-hDPSCs positively expressed the Beta-III tubulin, the microtubule element, to indicate the neuronal-like cell morphologies (Fig 5C, white arrows) that were consistently like the d-hDPSCs (Fig 5B, white arrows). Moreover, the neuronal morphologies of the d-hDPSCs and the ERGO-d-hDPSCs were presented in several shapes, including unipolar-like (Fig 5D), bipolar-like (Fig 5E), pyramidal-like (Fig 5F), and multipolar-like (Fig 5G), whereas the fibroblast-like morphology was highly detected in the Crt-hDPSCs group (Fig 5A, white asterisks). Interestingly, the co-treatment of ergothioneine (ERGO-d-hDPSCs) significantly promoted neuronal differentiation and reached the highest percentage of Beta-III tubulin-positive cells with neuronal-like shape, when compared to the Crt-hDPSCs and the d-hDPSCs, respectively (Fig 5H). Furthermore, the unipolar-like and bipolar-like shapes were highly observed at the ERGO-d-hDPSCs (Fig 5I).

### Neuronal stages of ergothioneine co-treatment

**Fig 6:**
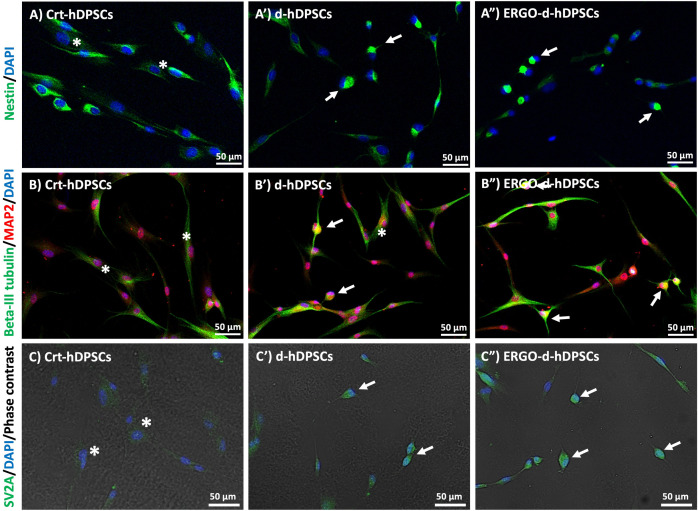
The immunophenotyping of neuronal-associated markers. **(A, A’, and A“)** The neuronal-like cells were positively expressed Nestin (Green, white arrows), and the undifferentiated cells revealed this expression with fibroblast-like morphology (green, white asterisks). **(B, B’, and B”)** Beta-III tubulin and MAP2 co-expression were investigated in the neuronal-like cells (white arrows) and were rarely detected in the undifferentiated cells (white asterisks). **(C, C’, and C”)** The phase contrast microscopy demonstrated the neuronal-like morphology. The neuronal-like cells from the d-hDPSCs and the ERGO-d-hDPSCs intensely revealed the SV2A expression (Green, white arrows), while the Crt-hDPSCs weakly detected the SV2A expression (Green, white asterisks). The nuclei were counter-stained with DAPI (blue). Scale bars: A, A’, A’‘, B, B’, B’‘, C, C’, and C” = 50 µm.

**Fig 7:**
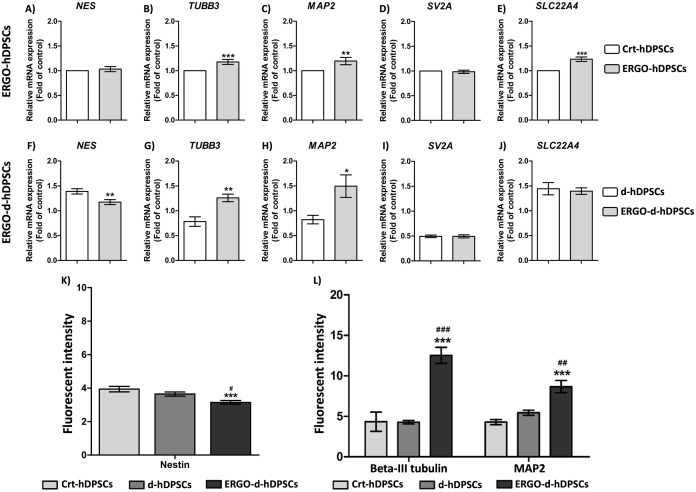
Gene expression and fluorescent intensity profiling. **(A-E)** The mRNA expression of neuronal-associated genes (*NES, TUBB3, MAP2, SV2A*, and *SLC22A4)* of the ergothioneine-treated cells (ERGO-hDPSCs) without neuronal induction medium was supplementary. The data were expressed as mean ± SEM and normalized to the control. **p-value < 0.01,***p-value < 0.001 when compared to the Crt-hDPSCs, the independent sample *t*-tests, n = 3. **(F-J)** The neuronal-associated genes profile of the ERGO-d-hDPSCs. The data were expressed as mean ± SEM and normalized to the control. *p-value < 0.05,**p-value < 0.01 when compared to the d-hDPSCs, the independent sample *t*-tests, n = 3. **(K)** The decreasing fluorescent intensity of early neuronal markers was observed at the ERGO-d-hDPSCs. **(L)** The increasing fluorescent intensity of late neuronal markers was observed at the ERGO-d-hDPSCs. The data were expressed as mean ± SEM, ***p-value < 0.001 when compared to the Crt-hDPSCs, ^#^*p*-value < 0.05, ^##^*p*-value < 0.01, ^###^*p*-value < 0.001 when compared to the d-hDPSCs, Tukey’s multiple comparison test, n = 3.

To investigate the neuronal stages of the neuronal-like cells, the typical markers of neuronal stages were performed. The neuronal-like cells of the d-hDPSCs and the ERGO-d-hDPSCs group positively expressed Nestin, which is the marker of NSCs (Fig 6A’ and 6A“, respectively, white arrows). However, the level of Nestin expression was slightly decreased and reached the lowest expression in the ERGO-d-hDPSCs (Fig 7K) and consistently with *NES* (mRNA that encodes for Nestin) expression (Fig 7F). Interestingly, the expression of Beta-III tubulin and MAP2, which are the late neuronal markers, was intensely expressed in the neuronal-like cells derived from the d-DPSCs and the ERGO-d-hDPSCs (Fig 6B’ and 6B’‘, respectively, white arrows) and demonstrated the highest fluorescent intensity when compared with the Crt-hDPSCs and the d-hDPSCs (Fig 7L). Moreover, the co-treatment of ergothioneine with neuronal induction medium significantly enhanced the mRNA expression of *TUBB3* (mRNA that encodes for Beta-III tubulin) and *MAP2,* resulting in high expression in the ERGO-d-hDPSCs (Fig 7G and 7H). These findings suggested that ergothioneine co-treatment may drive neuronal differentiation from the early to late stages.

Furthermore, without the neuronal induction medium, the ergothioneine (500 µM) treatment enhanced the expression of *TUBB3* (Fig 7B), *MAP2* (Fig 7C), and *SLC22A4* (mRNA that encodes for OCTN1) (Fig 7E) to indicate the potential internalized uptaking and promoting their neuronal differentiation of hDPSCs.

### The potential of functional neuronal activity profiling

To demonstrate the potential of functional neuronal activity within the neuronal-like cells, the expression of the SV2A, which is involved in the regulation of neurotransmitter release, was performed. The majority of neuronal-like cells from the d-hDPSCs and the ERGO-d-hDPSCs intensely expressed SV2A (Fig 6C’ and 6C“, respectively, white arrows). On the other hand, the SV2A expression was rarely observed in the Crt-hDPSCs group (Fig 6C, white asterisks). However, the *SV2A* expression, which was revealed by qRT-PCR, demonstrated no significant difference (Fig 7I).

**Fig 8:**
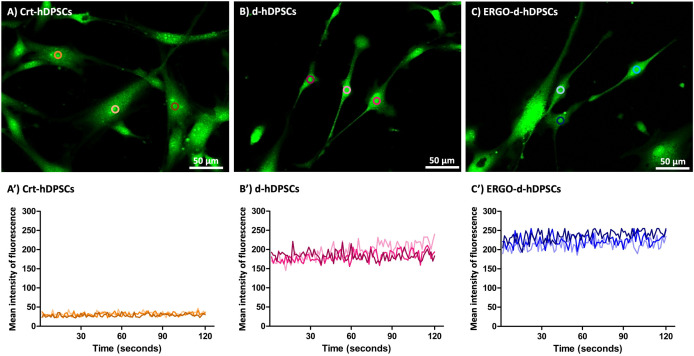
Neuronal activity. **(A-C)** Representative images of the Crt-hDPSCs, d-hDPSCs, and ERGO-d-hDPSCs were captured during intracellular calcium oscillation at 0 to 120 seconds, respectively. The Crt-hDPSCs expressed a weak fluorescent signal. The d-hDPSCs and ERGO-d-hDPSCs revealed a higher signal than the Crt-hDPSCs. **(A’)** The Crt-hDPSCs presented a low and narrow dynamic change of fluorescent intensity. **(B’-C’)** The higher and wider dynamic changes of fluorescent intensity were observed at d-hDPSCs and ERGO-d-hDPSCs, which indicated neuronal activity. Data are expressed as the mean intensity of fluorescence recorded for 120 seconds (n = 3). Scale bars: A-C = 50 µm.

The intracellular calcium oscillation activity was investigated to verify the functional neuronal activity and elucidate the activity of neurotransmitter release. The neuronal-like cells were activated by 50 mM KCI and observed the dynamic change of Ca^2+^ via Fluo-3 AM (Ca^2+^ indicator). The Crt-hDPSCs produced a weak fluorescent signal (Fig 8A) and presented a low and narrow dynamic change of fluorescent intensity (Fig 8A’). Interestingly, the d-hDPSCs (Fig 8B) and ERGO-d-hDPSCs (Fig 8C) revealed a higher signal than the Crt-hDPSCs. The higher and wider dynamic changes of fluorescent intensity were observed at d-hDPSCs and ERGO-d-hDPSCs, which indicated the potential of functional neuronal activity (Fig 8B’ and 8C’, respectively).

## Discussion

The dental-derived mesenchymal stem cells have been defined as promising candidates for stem cell-based therapy for neurological disorders [22]. The hDPSCs have great potential for a range of applications in stem cell research and regenerative medicine, including adipogenesis, osteogenesis, chondrogenesis, angiogenesis, dentinogenesis, and neurogenesis [23]. Importantly, these hDPSCs originated from ectomesenchyme, which is a derivative of migratory neural crest stem cells, the progenitor of neuronal cells in the nervous system [24]. Therefore, hDPSCs can differentiate into neuronal cells under specific conditions [25]. In this study, hDPSCs were established from human dental pulp tissue of third molars and performed an enzymatic digestion method to provide high proliferative ability [26]. The MSCs’ properties were characterized by plastic adherent ability, typical fibroblast-like morphology, multilineage differentiation ability, and cell surface antigen molecules profiling according to the minimal criteria provided by the International Society for Cell & Gene Therapy (ISCT) [9]. Additionally, the isolated cells demonstrated the positive expression of Nestin and Beta-III tubulin to confirm the ectomesenchyme origin [27]. Therefore, this study demonstrated the neuronal differentiation ability of hDPSCs.

Recently, developing strategies have been investigated to differentiate human MSCs into neuronal cells, including psychotropic drugs, small molecules, epigenetic modification, and enriched culture medium [28]. In this study, the neuronal differentiation ability of the hDPSCs was demonstrated by culturing the neuronal induction medium, which consisted of neurotrophic factor (bFGF) and stimulating chemical agents (DMSO, Beta-mercaptoethanol, and butylated hydroxyanisole). Importantly, these inducers play essential roles in activating neuronal morphology changes and neuronal differentiation [29]. The chemically enriched neuronal induction medium successfully promoted neuronal differentiation of hDPSCs, and the neuronal morphology and Beta-III tubulin expression were observed. Furthermore, the expression of neuronal-related genes, including *NES, NF-M*, and *Musashi-1* was highly expressed in neuronal-like cells [13]. Furthermore, under the administration of the neurogenic induction medium, the human stem cells from apical papilla (hSCAPs) were differentiated into neuronal-like cells, which positively expressed the Nissl substance and exhibited functional neuronal activity [30]. These results showed that the neuronal induction medium successfully induced neuronal differentiation of the MSCs.

The typical characteristics and specific parameters were elucidated to verify the characterization of *in vitro*-induced neuronal cells [31]. Firstly, the evaluation of cell morphology revealed that hDPSCs were triggered into the differentiated cells (d-hDPSCs, neuronal-like cells), which revealed the induced changes of cell morphology from fibroblast-like into neuronal-like shape under the neurogenic inducing environment. These neuronal-like cells exhibited elongated processes with round cell bodies that consistently presented their neuronal morphology with previous studies, including the neuronal cells derived from hDPSCs [12], hSCAPs [30,32], human stem cells derived from deciduous teeth (SHEDs) [33], and human bone marrow mesenchymal stem cells (hBM-MSCs) [34]. Consequently, the identification of the Nissl body was the essential technique to verify the typical hallmark of neurons according to its specificity. The Nissl substance is compact granules with rough endoplasmic reticulum and is revealed by Cresyl violet staining. The hippocampal formation, which is a neuronal cell compartment, demonstrated the Cresyl violet-positive cells [35]. The neuronal cells in the grey matter of the spinal cord positively represented the Nissl substance of neuronal cells [36]. Moreover, the *in vitro*-induced neuronal cells from hSCAPs [30], hDPSCs [12], and NSCs-derived hSCAPs [32,37] positively exhibited dark purple-stained Nissl bodies. These results indicated that the neuronal-like cells exhibited the expression of Nissl substance, as determined by Cresyl violet staining.

Furthermore, neurogenic-associated protein profiling was investigated to determine the stage of neuronal cells, indicated by immunofluorescent staining. Nestin is encoded by *NES*, which is expressed in NSCs (the early neuronal stage) [38]. The NSCs-derived hDPSCs under 3D-neurosphere induction exhibited the positive expression of Nestin [39]. Moreover, Beta-III tubulin is a microtubule element of the tubulin family that is predominantly found in neurons [40]. Immunofluorescent results demonstrated that the Beta-III tubulin-positive cells revealed their cytoskeleton to indicate several neuronal morphologies, including unipolar, bipolar, multipolar, and pyramidal shapes. MAP2 is a dendritically enriched protein and represents the markers of the late neurogenic/mature stage [41]. Additionally, SV2A is a transmembrane protein of synaptic vesicles, which was observed in synaptic terminals, demonstrating the regulation of neurotransmitter release activity [42]. These results demonstrated that the neuronal-like cells, which were induced with neuronal induction medium (positive control) were positively expressing neurogenic-associated proteins (Nestin, Beta-III tubulin, MAP2, and SV2A). Interestingly, without supplementary neuronal growth factors (negative control) presented that the undifferentiated cells can be observed expression of neurogenic-associated proteins, indicating their ectomesenchymal origin [27].

Recently, the potential of functional neuronal activities of neurons was demonstrated by the investigation of neuronal networks [43], intracellular signaling [44], and intercellular communication [45]. In this study, intracellular calcium oscillation was used to define the Ca^2+^ influx profiling, which plays an essential role during vesicular neurotransmitter-releasing activity [46]. To trigger neurotransmitter release, KCl treatment was performed to change the membrane potential and drive the action potential by intracellular calcium influx [47]. Moreover, the neuronal activity, represented by intracellular calcium oscillation was closely correlated to electrical activity recorded with the whole-cell patch clamp technique [48]. This study revealed that the dynamic change in fluorescent intensity of calcium signaling presented continuous high-intensity peaks and wide intervals at the differentiated neuronal-like cells (d-hDPSCs and ERGO-d-hDPSCs). In contrast, the hDPSCs were used as the negative control and exhibited low fluorescent intensity as a baseline pattern comparable to that demonstrated in a previous study [12]. Taken together, the hDPSCs were differentiated into neuronal cells, which presented the structural and functional characteristics of typical neuron profiles under neuronal-inducing conditions.

Neuronal cells are the functional units of the nervous system, which electrically exhibit signaling activity. Abnormalities of their structure, biological, and physiological activities lead to neurodegenerative diseases [49]. Importantly, regulation of adult neurogenesis and induction of neuronal differentiation is the essential approach in developing a therapy to cure and rescue neurodegenerative disease [14]. Therefore, an investigation focusing on enhancing neuronal differentiation ability using appropriate inducers could be performed. Ergothioneine is a natural compound, which exhibits hydrophilic properties and passes through the BBB by uptake via OCTN1 to indicate its potential in neurogenerative medicine [50]. It exerted beneficial effects on neurite outgrowth [51], neuronal differentiation [52], neuronal maturation [16], and protection against Beta-amyloid neurotoxicity [53]. Moreover, *in vivo* oral administration of ergothioneine significantly increased the number of newborn neurons (doublecortin-positive cells) in the hippocampal dentate gyrus [19].

This study demonstrated the enhancing effect of ergothioneine on the neuronal differentiation ability of hDPSCs and hypothesized that the appropriate concentration of ergothioneine could promote neuronal differentiation, resulting in a high number of neuronal cells. First, there was no cellular toxicity of ergothioneine on hDPSCs’ viability using the concentration test in these experiments. This result has clearly shown that ergothioneine treatment did not have a cytotoxic effect on the hDPSCs up to 500 μM for 30 hours. Moreover, IC_50_ > 500 μM is indicated as low cytotoxicity (IC_50_ more than 100 μM) [54]. Therefore, ergothioneine at concentrations 0–500 μM was investigated for the neuronal differentiation-enhancing potential on the hDPSCs. The highest outcome of the neuronal differentiation was observed at 500 μM (ERGO-d-hDPSCs), which is defined as the optimal concentration. Furthermore, the neurogenic maturation ability was triggered by co-treatment of ergothioneine with neuronal induction medium. The decreasing expression of the early neuronal stage markers (Nestin and *NES*) was revealed by immunofluorescent profiling and qRT-PCR, whereas the increasing expression of the late neuronal stage markers (Beta-III tubulin, *TUBB3,* and MAP2) was observed at the ERGO-d-hDPSCs, which indicates a shift in the neuronal stage. Our findings demonstrated the expression of OTCN1 (ergothioneine transporter) in the Crt-hDPSCs and neuronal-like cells. Interestingly, the administration of ergothioneine enhanced the *SLC22A4* expression. Taken together, the enhancing neuronal differentiation effect of ergothioneine on hDPSCs may be taken up through this transporter. However, further investigation of signaling pathways and the underlying mechanism of ergothioneine on neuronal differentiation after synergistically treating hDPSCs is still necessary to explore.

Consistently, treatment of 500 μM ergothioneine on mouse neural progenitor cells can suppress their proliferation, glial lineage differentiation, and specifically promote the differentiation into neuronal cells. The expression of the neuronal differentiation-related gene (*Math1*) was highly upregulated. Moreover, their study concluded that ergothioneine at 500 µM triggered neuronal differentiation of mouse neural stem cells (early stage) into neuronal cells (late stage), resulting in increasing the number of neuronal-like cells and the number of beta-III tubulin-positive cells. The results of immunofluorescent imaging and western blot analysis positively revealed the OCTN1 expression in mouse neural stem cells to indicate the potential internalized uptaking and promoting their neuronal differentiation. Furthermore, after knocking down this receptor with siRNA, their neuronal differentiation ability was suppressed [20].

Consistently, our findings demonstrated that co-treatment of ergothioneine at 500 µM enhanced neuronal differentiation of hDPSCs. The number of Cresyl violet-positive cells, the number of beta-III tubulin-positive cells, the mRNA expression of *TUBB3* and *MAP2* (late stage), and the fluorescent intensity of beta-III tubulin and MAP2 were increased, while the mRNA expression of *NES* (early stage) and the fluorescent intensity of Nestin were decreased. The qRT-PCR revealed that the *SLC22A4* was expressed in hDPSCs to indicate the potential internalized uptaking and promoting their neuronal differentiation of hDPSCs. Furthermore, the differentiated cells (d-hDPSCs) also expressed *SLC22A4,* and administration of 500 µM ergothioneine enhanced this *SLC22A4* expression in hDPSCs.

Taken together, the results of this study demonstrated the MSCs properties of hDPSCs and their neuronal differentiation ability. Administration of ergothioneine up to 500 μM for 30 hours did not trigger cytotoxicity of characterized hDPSCs. Importantly, co-treatment of ergothioneine 500 μM with the neuronal induction medium can enhance their neuronal differentiation ability, resulting in the high number of functional neuronal cells and triggering neurogenic maturation.

## Conclusions

This study demonstrates the neuronal differentiation ability of the hDPSCs. Interestingly, the co-treatment with ergothioneine at 500 µM enhances neuronal differentiation of the hDPSCs, resulting in the highest number of neuronal-like cells that presented structural and functional neuronal characteristics. Moreover, this optimal concentration has the potential to promote neurogenic maturation. Therefore, these findings suggest the alternative of using hDPSCs and the potential of ergothioneine co-treatment as stem cell-based therapy for further transplantation to cure various neurological diseases.
